# Acute infections and venous thromboembolism

**DOI:** 10.1111/j.1365-2796.2011.02473.x

**Published:** 2011-12-08

**Authors:** M Schmidt, E Horvath-Puho, R W Thomsen, L Smeeth, H T Sørensen

**Affiliations:** 1 Department of Clinical Epidemiology, Institute of Clinical Medicine, Aarhus University HospitalAarhus, Denmark; 2 Department of Non-Communicable Epidemiology, London School of Hygiene and Tropical MedicineLondon, UK

**Keywords:** antibacterial agents, deep venous thrombosis, infections, pulmonary embolism, venous thromboembolism

## Abstract

**Abstract:**

Schmidt M, Horvath-Puho E, Thomsen RW, Smeeth L, Sørensen HT (Department of Clinical Epidemiology, Institute of Clinical Medicine, Aarhus University Hospital, Aarhus, Denmark; and Department of Non-Communicable Epidemiology, London School of Hygiene and Tropical Medicine, London, UK). Acute infections and venous thromboembolism. *J Intern Med* 2012; **271**: 608–618.

**Background:**

Data on the association between acute infections and venous thromboembolism (VTE) are sparse. We examined whether various hospital-diagnosed infections or infections treated in the community increase the risk of VTE.

**Methods:**

We conducted this population-based case–control study in Northern Denmark (population 1.8 million) using medical databases. We identified all patients with a first hospital-diagnosed VTE during the period 1999–2009 (*n* = 15 009). For each case, we selected 10 controls from the general population matched for age, gender and county of residence (*n* = 150 074). We identified all hospital-diagnosed infections and community prescriptions for antibiotics 1 year predating VTE. We used odds ratios from a conditional logistic regression model to estimate incidence rate ratios (IRRs) of VTE within different time intervals of the first year after infection, controlling for confounding.

**Results:**

Respiratory tract, urinary tract, skin, intra-abdominal and bacteraemic infections diagnosed in hospital or treated in the community were associated with a greater than equal to twofold increased VTE risk. The association was strongest within the first 2 weeks after infection onset, gradually declining thereafter. Compared with individuals without infection during the year before VTE, the IRR for VTE within the first 3 months after infection was 12.5 (95% confidence interval (CI): 11.3–13.9) for patients with hospital-diagnosed infection and 4.0 (95% CI: 3.8–4.1) for patients treated with antibiotics in the community. Adjustment for VTE risk factors reduced these IRRs to 3.3 (95% CI: 2.9–3.8) and 2.6 (95% CI: 2.5–2.8), respectively. Similar associations were found for unprovoked VTE and for deep venous thrombosis and pulmonary embolism individually.

**Conclusions:**

Infections are a risk factor for VTE.

## Introduction

Any association between acute infection and venous thromboembolism (VTE) is of major clinical importance owing to the high rates of both conditions [1]. VTE affects more than 1 in a 1000 individuals each year in Western populations [2–4]. It occurs predominantly in the deep vessels of the lower limbs (i.e. deep venous thrombosis; DVT) [2–4], with a risk of serious complications such as pulmonary embolism (PE) and post-thrombotic syndrome [2, 4]. Acute infection and the associated systemic inflammation may increase the risk of VTE [1, 5] owing to one or more of the three precipitants of venous thrombosis proposed by Virchow: venous stasis, increased coagulability and vessel wall damage [6, 7].

Available evidence for an association between acute infection and VTE is limited [1, 8–10], and no studies have been conducted to examine the magnitude and duration of increased VTE risk associated with various infections either diagnosed in hospital or treated in the community. Therefore, we examined whether patients with hospital-diagnosed or community-treated infections were at increased risk of subsequent VTE.

## Materials and methods

### Setting

This study was conducted in northern Denmark, an area with 1.8 million inhabitants (approximately 30% of the Danish population). Since the beginning of 1998, complete computerized prescription records have been available for this population. Our study period began on 1 January 1999, thus providing at least 1 year of prescription history for all study participants and ended on 31 December 2009.

The Danish National Health Service provides universal tax-supported healthcare, guaranteeing unrestricted access to primary and secondary care, free at the point of delivery and providing partial reimbursement for prescribed medications, including antibiotics [11]. Accurate and unambiguous linkage of all registries at the individual level is possible in Denmark using the unique central personal registry number assigned to each Danish resident at birth or upon immigration [12].

### Venous thromboembolism

We used the Danish National Registry of Patients (DNRP) [13], covering all Danish hospitals, to identify all patients in the region with a first VTE, defined by an incident inpatient or outpatient (clinic or emergency room visit) diagnosis of lower limb DVT or PE, during the study period. The DNRP contains data on dates of inpatient admission and discharge from nonpsychiatric hospitals since 1977 and of emergency room and hospital outpatient clinic visits since 1995 [13]. Each discharge is associated with one primary diagnosis and one or more secondary diagnoses classified according to the International Classification of Diseases, 8th revision (ICD-8) until the end of 1993 and the 10th revision (ICD-10) thereafter [13].

We identified both primary and secondary diagnoses of DVT and PE in the DNRP. Patients coded as having both DVT and PE were classified as PE patients. To reduce the impact of potential coding errors, we excluded patients with an incident outpatient diagnosis of PE, but without a subsequent inpatient diagnosis of any VTE. In a secondary analysis, we focused on cases with unprovoked VTE by excluding patients with the following classical risk factors: pregnancy, major trauma, fracture or surgery within 3 months preceding VTE, and pre-existing cancer or a new cancer diagnosis within 3 months after VTE [14]. The date of the first hospital diagnosis of a VTE was considered the index date.

### Population controls

We used the Danish Civil Registration System to select 10 population controls for each case, matched for age (birth year), gender and county of residence [12]. This registry has maintained data on all vital statistics – including date of birth, change(s) of address, date of emigration and exact date of death – for the Danish population since 1968 [12]. We selected controls using risk-set sampling: each control had to be alive and at risk of a first VTE hospitalization on the index date of the case to whom he/she was matched [15]. For three cases over 100 years of age, 10 controls with an exact match could not be found. Controls were assigned an index date identical to that of each corresponding case.

### Hospital and community infections

The DNRP [13] was used to identify patients with in- or outpatient hospital contact because of specific infections before their index date. We examined the VTE risk associated with the following most frequently hospital-diagnosed types of infection: systemic respiratory tract infection (RTI; defined as pneumonia, acute bronchitis or influenza), urinary tract infection (UTI), skin infection (including cellulitis and erysipelas), intra-abdominal infection (including gastrointestinal infection) and septicaemia [16].

We used the regional prescription database [17] to identify all antibiotic prescriptions filled by cases and controls 1 year before their index date. In Denmark, antibiotics are only available by prescription. Pharmacies in Denmark are equipped with electronic accounting systems, primarily used to secure reimbursement from the National Health Service. For each filled prescription, patient information regarding the central personal registry number, the type and amount of drug prescribed according to the Anatomical Therapeutic Chemical (ATC) classification system and the date the drug was dispensed are transferred from the pharmacy to the prescription database [17]. We identified the following antibiotic groups, which together account for more than 90% of the total community antibiotic sales in Denmark [11]: penicillins, cephalosporins, macrolides, quinolones, tetracyclines and sulphonamides. We categorized these antibiotics according to the community-associated infection types for which they are mainly indicated in Denmark [18]: RTIs, UTIs, skin or soft-tissue infections and infections treated with focus-unspecific penicillins. The associated ICD and ATC codes are provided in Table S1.

### Patient characteristics

We obtained information from the DNRP [13] on inpatient (since 1977) and outpatient (since 1995) diagnoses of the following potential confounding conditions: cardiovascular disease, chronic obstructive pulmonary disease (COPD) or asthma, diabetes, liver disease, obesity, osteoporosis and renal failure. To account further for potential unmeasured confounding arising from frailty and immobility, we created the variable ‘another recent inpatient admission’, which includes any inpatient diagnoses other than the infectious and comorbid diagnoses listed previously within 3 months before the index date. To ensure complete identification of subjects with diabetes, COPD or cardiovascular disease, we used the prescription database to obtain data on ever use of the following drugs since 1998: antidiabetic agents (oral antidiabetic drugs and insulin) and medicines for respiratory diseases and cardiovascular diseases (angiotensin-converting enzyme inhibitors or angiotensin-II receptor inhibitors, aspirin, beta-blockers, calcium channel blockers, clopidogrel, diuretics, nitrates, statins and other antihypertensive agents). We also obtained data on concurrent use of nonsteroidal anti-inflammatory drugs, oral glucocorticoids, postmenopausal hormone-replacement therapy and vitamin K antagonists because these drugs affect the risk of VTE [3, 4, 19]. Associated ICD codes, ATC codes and exposure windows for co-medications are provided in Table S1.

### Statistical analysis

We first created contingency tables for the main study variables, from which we calculated the numbers and proportions of cases and controls in exposure, medical and demographic subcategories. We then stratified the contingency tables according to each of the potential confounding factors listed in Table 1 [20]. Next, we used conditional logistic regression to compute odds ratios (ORs) with 95% confidence intervals (CIs) for VTE, comparing patients with hospital-diagnosed infection and/or antibiotic treatment in the community within 1 year before their index date with the reference group of subjects with no hospital-diagnosed infection or filled community antibiotic prescription within the same period. In the primary analysis, we examined the OR for VTE within the first 3 months after infection. Because we used risk-set sampling of controls, the ORs estimate the incidence rate ratios (IRRs) [21]. We then fitted models adjusting for the potential confounding factors listed in Table 1, that is, age (<55, 55–70 and ≥71 years), gender, the classical VTE risk factors, other comorbidities, another recent inpatient admission and co-medications. We repeated the analyses for: (i) RTI, UTI, skin infection, intra-abdominal infection and septicaemia, as defined by hospital diagnoses, (ii) RTI, UTI, skin or soft-tissue infection and infections treated with focus-unspecific penicillins, as defined by filled antibiotic prescriptions, (iii) VTE registered only as the primary diagnosis, (iv) unprovoked VTE, and (v) DVT and PE individually.

**Table 1:** Characteristics of cases with venous thromboembolism (VTE) and population controls

	All VTE		Unprovoked VTE	
	
	Cases (%) *n* = 15 009	Controls (%) *n* = 150 074	Cases (%) *n* = 9113	Controls (%) *n* = 79 061
Age				
<55 years	4294 (28.6)	42 940 (28.6)	2940 (32.3)	27 423 (34.7)
55–70 years	4556 (30.4)	45 560 (30.4)	2619 (28.7)	23 226 (29.4)
≥71 years	6159 (41.0)	61 574 (41.0)	3554 (39.0)	28 412 (35.9)
Median age (IQR)	63.5 (52.0–77.0)	63.4 (52.0–77.0)	62.2 (49.0–77.0)	61.1 (48.0–76.0)
Gender, female	7947 (52.9)	79 463 (52.9)	4738 (52.0)	40 461 (51.2)
Classical VTE risk factors				
Cancer^a^	3247 (21.6)	13 088 (8.7)	–	–
Pregnancy^b^	111 (0.7)	371 (0.2)	–	–
Surgery^b^	3341 (22.3)	7307 (4.9)	–	–
Trauma or fracture^b^	1164 (7.8)	2803 (1.9)	–	–
Other comorbidities				
Cardiovascular disease^d^	9080 (60.5)	72 790 (48.5)	5229 (57.4)	34 791 (44.0)
COPD or asthma^d^	3803 (25.3)	25 234 (16.8)	2293 (25.2)	12 409 (15.7)
Diabetes^d^	1170 (7.8)	9124 (6.1)	652 (7.2)	4275 (5.4)
Liver disease^c^	238 (1.6)	850 (0.6)	136 (1.5)	383 (0.5)
Obesity^c^	773 (5.2)	3096 (2.1)	434 (4.8)	1511 (1.9)
Osteoporosis^c^	511 (3.4)	3383 (2.3)	272 (3.0)	1448 (1.8)
Renal failure^c^	318 (2.1)	1043 (0.7)	137 (1.5)	409 (0.5)
Another recent inpatient admission^e^	3827 (25.5)	6148 (4.1)	1110 (12.2)	1364 (1.7)
Co-medications^f^				
HRT	693 (4.6)	6409 (4.3)	410 (4.5)	2985 (3.8)
NSAIDs	2528 (16.8)	10 041 (6.7)	1465 (16.1)	4947 (6.3)
Oral glucocorticoids	1455 (9.7)	3541 (2.4)	717 (7.9)	1527 (1.9)
Vitamin K antagonists	461 (3.1)	3436 (2.3)	221 (2.4)	1470 (1.9)

COPD, chronic obstructive pulmonary disease; HRT, postmenopausal hormone-replacement therapy; IQR, interquartile range; NSAID, nonsteroidal anti-inflammatory drug.

a Pre-existing cancer or a cancer diagnosis within 3 months after the VTE.

b Any in- or outpatient diagnosis within 3 months before the VTE.

c Any in- or outpatient hospital diagnosis since 1977.

d Any in- or outpatient diagnosis since 1977 or any filled prescription for the disease since 1998.

e Any inpatient diagnosis within 3 months before the VTE, other than for the risk factors and comorbidities listed.

f Prescription filled within 60 days (NSAIDs and oral glucocorticoids) or 90 days (HRT and vitamin K antagonists) before the VTE.

To examine the impact of time from onset of infection (date of diagnosis or prescription redemption) and VTE occurrence, we repeated the analysis for different postinfection risk periods, that is, time intervals between the last hospital diagnosis and/or antibiotic prescription redemption and VTE: 0–2, 3–4, 5–8, 9–12, 13–26, 27–39 and 40–52 weeks. We used the Wald chi-square test to test for a correlation in time between onset of infection and VTE occurrence.

Stratified analyses of the association between any infection (hospital-diagnosed infection or infection treated in the community within 3 months before VTE) and VTE were conducted using predefined subgroups based on age (<55, 55–70 and ≥71 years), gender, presence/absence of cancer, trauma, fracture, diabetes, cardiovascular disease, COPD or asthma, obesity or another recent inpatient admission and the total number of antibiotic prescriptions within 365 days before the index date (1–5, 6–10 or >10 prescriptions) [1].

Because diagnoses of VTE recorded in the emergency room have a low positive predictive value (approximately 30%) [22], we performed a sensitivity analysis excluding such diagnoses. Finally, we quantified the influence of potential unmeasured confounding on the observed association by means of a rule-out approach [23]. We estimated how strongly a single unmeasured binary confounder would need to be associated with infection and VTE to fully explain our findings and illustrated this association graphically (Figure S1). We assumed as a worst-case scenario that the prevalence of such an unmeasured confounder was 30% and that 12% of the population had an infection within 3 months (as observed for the controls).

## Results

### Patient characteristics

We identified 15 009 patients with VTE and 150 074 population controls. Amongst the VTE cases, 13 259 (88.3%) had an inpatient hospital diagnosis of VTE, 1080 (7.2%) had a hospital outpatient clinic diagnosis and 670 (4.5%) were diagnosed during an emergency room visit. Slightly more than half of the cases with VTE were women, and the median age was 64 years (Table 1). Within 3 months before the admission of VTE cases, 1062 cases (7.1%) and 1304 controls (0.9%) had a hospital-diagnosed infection, and 4356 cases (29.0%) and 16 693 controls (11.1%) filled an antibiotic prescription in the community. Compared with controls, many more VTE patients had cancer, trauma, surgery or another inpatient admission, or were pregnant, within 3 months before VTE. Other comorbidities, as well as use of co-medications, were also more common amongst cases than controls. Amongst all VTE patients, 9113 (60.7%) appeared to have unprovoked VTE. The distribution of characteristics was similar amongst patients with unprovoked VTE and the overall group (Table 1).

### Risk of VTE

Compared with subjects with no infections during the year before VTE, the IRR for VTE was 12.5 (95% CI: 11.3–13.9) for patients with hospital-diagnosed infection and 4.0 (95% CI: 3.8–4.1) for patients treated with antibiotics in the community (Table 2). Adjusting for confounders reduced these IRR values to 3.3 (95% CI: 2.9–3.8) and 2.6 (95% CI: 2.5–2.8), respectively. The covariate with the most influence was another recent inpatient admission.

**Table 2:** Incidence rate ratios for venous thromboembolism (VTE) associated with hospital-diagnosed infections and infections treated in the community

	Incidence rate ratio (95% confidence intervals)					
	
	All VTE			Unprovoked VTE		
	
	No. of cases/controls	Unadjusted^a^	Adjusted^b^	No. of cases/controls	Unadjusted^a^	Adjusted^b^
No infection^c^	7088/103 370	1 (reference)	1 (reference)	4684/56 236	1 (reference)	1 (reference)
Infection, overall^d^	4836/17 367	4.2 (4.1–4.4)	2.7 (2.5–2.8)	2595/7909	4.0 (3.8–4.2)	3.0 (2.8–3.2)
Hospital-diagnosed infection^d^	1062/1304	12.5 (11.3–13.9)	3.3 (2.9–3.8)	390/360	13.7 (11.3–16.5)	5.0 (4.0–6.2)
Respiratory tract infection	561/499	17.4 (14.8–20.4)	4.9 (4.1–5.9)	248/166	20.5 (15.7–26.8)	7.7 (5.7–10.4)
Urinary tract infection	243/414	8.9 (7.3–10.8)	1.7 (1.4–2.2)	91/109	8.7 (6.1–12.2)	2.0 (1.3–2.9)
Skin infection	145/171	12.8 (9.7–16.8)	4.1 (3.0–5.7)	57/43	14.2 (8.9–22.6)	6.2 (3.7–10.5)
Intra-abdominal infection	147/212	10.5 (8.1–13.7)	2.4 (1.8–3.3)	29/39	9.2 (5.1–16.6)	3.1 (1.5–6.3)
Septicaemia	98/81	18.9 (12.8–28.1)	3.6 (2.3–5.8)	19/18	13.3 (5.8–30.6)	4.9 (1.9–12.7)
Community antibiotic treatment^d^	4356/16 693	4.0 (3.8–4.1)	2.6 (2.5–2.8)	2445/7731	3.9 (3.6–4.1)	3.0 (2.8–3.1)
Antibiotics for respiratory tract infection	939/2808	5.2 (4.8–5.7)	3.4 (3.0–3.7)	539/1332	5.2 (4.6–5.8)	3.6 (3.2–4.1)
Antibiotics for urinary tract infection	1431/6630	3.4 (3.2–3.7)	2.0 (1.8–2.1)	722/2894	3.1 (2.8–3.4)	2.1 (1.9–2.4)
Antibiotics for skin or soft tissue infection	615/1327	7.2 (6.4–8.1)	3.8 (3.3–4.4)	312/524	7.5 (6.3–8.9)	5.6 (4.7–6.7)
Focus-unspecific penicillins	2266/7636	4.4 (4.2–4.7)	3.1 (2.9–3.3)	1361/3670	4.6 (4.2–4.9)	3.6 (3.3–3.9)

a Age-, gender-, and county-matched conditional logistic regression.

b Adjusted for the classical VTE risk factors, other comorbidities, another recent hospital admission and co-medications use, as listed in Table 1. Classical risk factors were not included, per definition, in the model for unprovoked VTE.

c No hospital-diagnosed infection or filled community antibiotic prescription within 365 days before the VTE.

d In- or outpatient hospital-diagnosed infection and/or filled community antibiotic prescription within 3 months before the VTE.

Amongst hospital-diagnosed infections, the adjusted IRR for subsequent VTE was 4.9 (95% CI: 4.1–5.9) for RTI, 1.7 (95% CI: 1.4–2.2) for UTI, 4.1 (95% CI: 3.0–5.7) for skin infection, 2.4 (95% CI: 1.8–3.3) for intra-abdominal infection and 3.6 (95% CI: 2.3–5.8) for septicaemia. Amongst infections treated with antibiotics in the community, the adjusted IRRs were similar: 3.4 (95% CI: 3.0–3.7) for RTI, 2.0 (95% CI: 1.8–2.1) for UTI, 3.8 (95% CI: 3.3–4.4) for skin or soft-tissue infection and 3.1 (95% CI: 2.9–3.3) for infections treated with focus-unspecific penicillins.

The effect estimates were increased similarly for patients with VTE recorded as the primary diagnosis (Table S2>), for patients with DVT and PE (Table S3) and for VTE patients diagnosed in settings other than the emergency room (Table S4). IRRs were even higher (Table 2) for unprovoked VTE [adjusted IRR values were 5.0 (95% CI: 4.0–6.2) for patients with hospital-diagnosed infection, 3.0 (95% CI: 2.8–3.1) for patients treated with antibiotics in the community and 3.0 (95% CI: 2.8–3.2) overall].

There was a correlation (*P* < 0.0001) in time between onset of infection and VTE occurrence: the highest VTE risk estimates were observed within the first 2 weeks after onset of all infections, declining gradually thereafter (Table 3). The incidence rate of VTE was increased eightfold within the first 2 weeks after hospital-diagnosed infection, with the highest risk increases associated with RTIs and skin infections. VTE rates were increased three- to four-fold between 3 and 8 weeks after infection, and then remained approximately twofold higher for almost all types of infection for up to 1 year of follow-up. For antibiotic-treated infections in the community, the rate of VTE was increased 5.5-fold within the first 2 weeks. The highest risk increases were associated with antibiotics typically used for RTIs and skin infections, in agreement with the findings for hospital-diagnosed infections. VTE risk estimates gradually declined to an increase of two- to three-fold within 3–8 weeks following most types of infections and remained 1.2- to 1.6-fold higher after 1 year of observation.

**Table 3:** Impact of the post-infection risk period on the association between infection and venous thromboembolism (VTE)

	Adjusted incidence rate ratio (95% confidence interval)^a^							
		
	Post-infection risk period^b^							
		
	0–2 weeks	3–4 weeks	5–8 weeks	9–12 weeks	13–26 weeks	27–39 weeks	40–52 weeks	*P*-value^c^
Infection, overall	5.6 (5.2–6.0)	2.5 (2.3–2.7)	1.9 (1.7–2.0)	1.5 (1.4–1.7)	1.4 (1.3–1.5)	1.2 (1.1–1.3)	1.2 (1.1–1.3)	<0.0001
Hospital-diagnosed infection	8.0 (6.4–10.0)	4.1 (3.2–5.3)	2.8 (2.3–3.4)	1.9 (1.5–2.4)	2.3 (2.0–2.7)	2.1 (1.7–2.5)	2.0 (1.6–2.4)	<0.0001
Respiratory tract infection	12.9 (8.7–19.1)	5.0 (3.3–7.5)	4.1 (2.9–5.6)	2.8 (1.9–4.2)	3.0 (2.3–3.8)	2.3 (1.7–3.0)	1.8 (1.3–2.5)	<0.0001
Urinary tract infection	3.5 (2.1–5.7)	2.3 (1.4–3.9)	1.8 (1.2–2.7)	1.0 (0.6–1.6)	2.2 (1.6–2.9)	2.2 (1.5–3.0)	2.0 (1.4–3.0)	0.02
Skin infection	12.2 (6.5–23.2)	8.7 (3.8–20.1)	1.0 (0.5–1.8)	3.2 (1.7–6.3)	3.0 (2.0–4.6)	3.1 (2.0–4.9)	2.1 (1.3–3.5)	<0.0001
Intra-abdominal infection	5.7 (2.8–11.9)	2.1 (1.0–4.3)	1.9 (1.2–3.2)	1.8 (0.9–3.4)	1.5 (1.0–2.3)	1.9 (1.1–3.0)	1.9 (1.2–3.1)	0.12
Septicaemia	8.7 (3.2–23.7)	4.9 (1.5–16.1)	2.6 (1.1–5.9)	1.9 (0.8–4.3)	2.2 (1.2–4.0)	1.4 (0.6–2.9)	1.3 (0.5–3.0)	0.06
Community antibiotic treatment	5.5 (5.1–5.9)	2.3 (2.1–2.6)	1.8 (1.7–2.0)	1.6 (1.4–1.7)	1.4 (1.3–1.5)	1.2 (1.1–1.3)	1.2 (1.1–1.3)	<0.0001
Antibiotics for respiratory tract infection	8.0 (6.6–9.6)	2.9 (2.3–3.6)	2.4 (2.0–2.9)	2.0 (1.6–2.5)	1.5 (1.3–1.7)	1.4 (1.2–1.7)	1.4 (1.2–1.6)	<0.0001
Antibiotics for urinary tract infection	2.7 (2.4–3.1)	1.9 (1.6–2.3)	1.7 (1.5–1.9)	1.7 (1.5–2.0)	1.4 (1.3–1.6)	1.4 (1.2–1.5)	1.4 (1.3–1.6)	<0.0001
Antibiotics for skin or soft tissue infection	10.7 (8.4–13.7)	3.1 (2.2–4.2)	1.8 (1.3–2.3)	2.5 (1.9–3.3)	1.9 (1.6–2.2)	1.8 (1.5–2.3)	1.6 (1.3–1.9)	<0.0001
Focus-unspecific penicillins	8.0 (7.2–9.0)	2.8 (2.5–3.3)	2.2 (2.0–2.5)	1.6 (1.4–1.8)	1.5 (1.4–1.6)	1.3 (1.2–1.5)	1.3 (1.1–1.4)	<0.0001

a Computed with conditional logistic regression adjusted for the classical VTE risk factors, other comorbidities, another recent hospital admission and co-medication use, as listed in Table 1. The reference group had no hospital-diagnosed infection or community antibiotic prescription redemption within 365 days before the VTE.

b The time interval between onset of infection and VTE occurrence.

c Wald χ^2^ test for no correlation in the adjusted model.

**Fig. 1:**
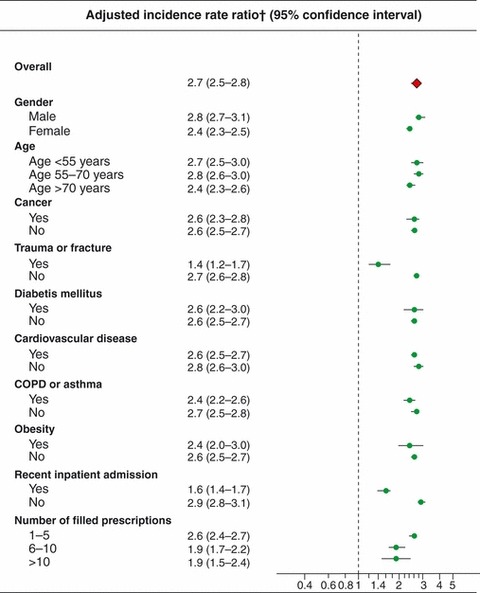
Stratified analysis of the association between infection and venous thromboembolism (VTE). In- or outpatient hospital-diagnosed infection and/or filled antibiotic prescription within 3 months before the VTE. Patients without hospital-diagnosed infection or filled community antibiotic prescription within 365 days before the VTE comprised the reference group within each category. *Adjusted for the classical risk factors, other comorbidities, another recent hospital admission and co-medications use, as listed in *Table 1*.

The effect estimates for VTE associated with infection remained elevated in all subgroups Figure 1. However, the effect estimates were modified by gender, with a slightly lower IRR amongst female [2.4 (95% CI: 2.3–2.5)] than male subjects [2.8 (95% CI: 2.7–3.1)]. Consistent with the assumption that the effect estimates would be lower amongst those at higher baseline risk, the estimates were lower amongst patients with [1.4 (95% CI: 1.2–1.7)] than without [2.7 (95% CI: 2.6–2.8)] trauma or fracture within 3 months before VTE and amongst patients with [1.6 (95% CI: 1.4–1.7)] compared with those without [2.9 (95% CI: 2.8–3.1)] another recent inpatient admission. Shorter length of antibiotic treatment (1–5 filled prescriptions) was associated with higher effect estimates than prolonged treatment (>5 filled prescriptions). There were no substantial differences in IRR amongst age subgroups and patients with or without cancer, diabetes, cardiovascular disease, COPD or asthma or obesity.

We estimated that an unmeasured confounder with a sevenfold higher prevalence amongst patients with infection than amongst those without infection would need to increase the VTE risk by a factor of 20 or more to fully explain our findings, if no increased risk actually existed (Figure S1). Even stronger confounders would be required to explain the findings for unprovoked VTE.

## Discussion

In this population-based case–control study of 15 009 patients with VTE, we found that acute infections diagnosed in hospital or treated in the community were associated with a markedly increased risk of VTE. The association was strongest within the first 2 weeks after infection onset and gradually declined thereafter. Similar associations were consistently found for unprovoked VTE, DVT and PE individually, and a variety of infection types were associated with VTE independent of whether they occurred in the hospital or community setting. We also found that any inpatient admission within 3 months for conditions other than the classical risk factors for VTE was strongly associated both with infection and VTE and modified the effect of infection on VTE occurrence.

This is the first study to examine the association between acute infection and VTE, distinguishing between infections diagnosed in hospital versus community care settings, and between various types of infection. In addition to community-diagnosed RTIs and UTIs [1], the association also held for skin and soft-tissue infections diagnosed in the community, as well as hospital-diagnosed RTIs, UTIs, skin infections, intra-abdominal infections and septicaemia. Initial evidence for an association between infection and VTE was obtained from studies showing a protective effect on VTE risk of thromboprophylaxis amongst patients with infectious disease [24–28]. A case–control study, including 636 DVT patients diagnosed in general practices between 1990 and 1991 in France, found that the adjusted odds for DVT were 1.95-fold (95% CI: 1.31–2.92) higher amongst patients with infectious disease compared with those without [9]. In another study including 866 acutely ill and immobilized inpatients, the adjusted OR for VTE within 14 days after infection was 1.74 (95% CI: 1.12–2.75) [8]. The type of infection was not described in either of these studies [8, 9]. A necropsy review from the UK showed that amongst patients with fatal PE, 12.2% (26 of 214) of those who had not undergone recent surgery had experienced an acute infectious episode in the 6 weeks before death [29]. A case series of 7278 DVT and 3755 PE patients using data from UK general practices between 1987 and 2004 found a twofold increased IRR for both DVT and PE within the first 2 weeks after UTI, a twofold increased IRR for DVT and 11-fold increased IRR for PE within the first 2 weeks after RTI (the later result presumed biased); the IRR gradually returned to baseline after 1 year [1]. The association between RTI and VTE was examined in another case–control study using data on 11557 DVT and 5162 PE patients who were included in a primary care database in the UK between 1991 and 2006 [10]. By excluding patients with an RTI within 1 month before PE, that study attempted to avoid possible misdiagnoses of early PE that may have biased previous studies [1]. Supporting our results, this last study found an increased risk of PE between 5 and 12 weeks after RTI (adjusted OR, 2.50, 95% CI: 1.33–4.72), an increased risk of DVT within 4 weeks after infection (adjusted OR, 2.64, 95% CI: 1.62–4.29) and increased risks of both PE and DVT for up to 1 year after the RTI [10].

Both the early and prolonged associations with VTE were strongest amongst patients with a hospital-based diagnosis. The higher increase in the early risk of VTE may be caused by more severe inflammation and immobilization associated with hospitalized compared with community-treated infection episodes, whereas some of the more sustained risk increase amongst patients with hospital-diagnosed infections may be due to uncontrolled higher baseline risk of VTE compared with patients treated in the community.

Venous thromboembolism may also be triggered by infection-associated systemic inflammation [1, 5]. We observed a more pronounced association between subsequent VTE and RTIs and skin infections typically caused by Gram-positive bacteria such as pneumococci, staphylococci and streptococci, compared with UTIs and intra-abdominal infections. Gram-positive infections may be associated with a more severe and early inflammatory response than typical Gram-negative infections such as UTIs and intra-abdominal infections [30]. This may be important with regard to our finding of higher VTE risk estimates for Gram-positive bacterial infections [30].

The population-based design in the setting of a universal single-payer healthcare system largely eliminated selection bias stemming from selective inclusion of specific hospitals, health insurance systems or age groups. Because antibiotic treatment was a marker for infection, potential nonadherence to prescribed medication did not influence the results. Furthermore, we were able to identify the period of increased risk following infection. The data on classical risk factors that defined provoked VTE have high validity and specificity [31].

A potential weakness is that our VTE data were derived from discharge registry diagnoses, which may have only 80% validity [22]. However, our results remained unchanged when we restricted the analysis to primary VTE diagnoses and excluded emergency room VTE diagnoses. Another concern is that individuals with infection in the community were only included in the analysis if they consulted their general practitioner. Also, the date of diagnosis or prescription filling may not correspond to the actual date of onset of VTE or infection. However, most patients visit their general practitioner within 3 days of onset of symptoms [32], so any underestimation of the duration of the increased risk of VTE was limited to a few days. In any case, any misclassification of VTE diagnosis, inaccuracy in onset date or incomplete ascertainment of infectious diseases would most likely be nondifferential, leading to underestimates of effect size.

Early presentation of PE may be misdiagnosed as an RTI, producing a misleadingly strong association between RTI and subsequent PE. The same may be true for DVT and skin infections. However, such diagnostic misclassification would be unlikely to account for the increased risks, observed after the first 2 weeks following infection onset, of DVT after RTI, as well as of PE after UTI, skin infection, intra-abdominal infection or septicaemia.

Although we were able to control for substantial confounding, our results may be vulnerable to confounding from unmeasured variables, including use of oral contraceptives, smoking and body mass index [2–4]. Because infection was associated with VTE amongst both women and men, oral contraceptive use is unlikely to have had a substantial confounding influence. We adjusted for unmeasured lifestyle factors by controlling for history of COPD and ischaemic heart disease. Finally, our results could not be explained by even a strong single unmeasured confounder. In conclusion, we found that all types of acute infections are strong predictors of subsequent VTE.

## Supporting Information

**Figure S1.** Required strength of an unmeasured confounder.

**Table S1.** ICD and ATC codes.

**Table S2.** Incidence rate ratios for venous thromboembolism recorded as primary diagnosis associated with hospital-diagnosed infections and infections treated in the community.

**Table S3.** Incidence rate ratios for infection associated with deep venous thrombosis or pulmonary embolism.

**Table S4.** Incidence rate ratios for infection associated with venous thromboembolism diagnosed in settings other than the emergency room.
