# Supplementary material for: Acute infections and venous thromboembolism
Source: J Intern Med. 2011 Dec 8;271(6):608–18. doi: 10.1111/j.1365-2796.2011.02473.x (PMC3505369; doi:10.1111/j.1365-2796.2011.02473.x)
Supplement: Supplementary file 1 [file joim0271-0608-SD1.docx]

**eFigure 1. Required strength of an unmeasured confounder**

Sensitivity analysis illustrating how strongly an unmeasured confounder would need to be associated with infectious disease (prevalence ratio for exposure–confounder association; PR_EC_) and VTE (relative risk of the disease in patients with the confounder; RR_CD_) to fully explain our estimates. The graphs depict the adjusted incidence rate ratio (IRR) for VTE associated with infectious disease overall (solid line), along with the lower limit of the 95% confidence interval (dashed line).
