# Supplementary material for: Acute infections and venous thromboembolism
Source: J Intern Med. 2011 Dec 8;271(6):608–18. doi: 10.1111/j.1365-2796.2011.02473.x (PMC3505369; doi:10.1111/j.1365-2796.2011.02473.x)
Supplement: Supplementary file 2 [file joim0271-0608-SD2.docx]

| **eTable 1: ICD and ATC codes** | |
| --- | --- |
| **Venous thromboembolism** |  |
| Deep venous thromboembolism (DVT) | ICD-8: 451.00; ICD-10: I80.1-3 |
| Pulmonary embolism (PE) | ICD-8: 450.99; ICD-10: I26 |
| **Hospital-diagnosed infectious diseases** |  |
| Candidiasis and other fungal infections | ICD-10: B35-B49 |
| CNS infections (except meningococcal disease) | ICD-10: G00-G07 |
| Female pelvic infections | ICD-10: N70-N77 |
| Gastrointestinal and intra-abdominal infections | ICD-10: A00-A09; K35, K37, K57.0, K57.2, K57.4, K57.8, K61, K63.0, K65.0, K65.9, K67, K75.0, K75.1, K80.0, K80.3, K80.4, K81.0, K81.9, K83.0 |
| Cardiac infections | ICD-10: I00-I02, I30.1, I32.0, I33, I38, I40.0 |
| Herpes simplex or zoster | ICD-10: B00-B02 |
| HIV | ICD-10: B20-B24 |
| Male genital infections | ICD-10: N41, N45 |
| Meningococcal disease | ICD-10: A39 |
| Miscellaneous bacterial infections | ICD-10: A20-A38, A42-A44, A48-A49, A65-A79 |
| Miscellaneous viral infections | ICD-10: A80-A99, B03-B09, B25-B34 |
| Parasitic infections | ICD-10: B50-B89 |
| Respiratory tract infections (systemic) | ICD-10: J09-J18, -22 |
| Septic arthritis, osteomyelitis | ICD-10: M00, M01, M86 |
| Septicaemia | ICD-10: A40-A41 |
| Sexually transmitted diseases | ICD-10: A50-A64 |
| Skin infections, including cellulitis/erysipelas | ICD-10: A46, L00-L03, L05-L08 |
| Tuberculosis | ICD-10: A15-A19 |
| Upper respiratory tract infections | ICD-10: J00-J06, J36, J39.0 |
| Urinary tract infections | ICD-10: N10, N12, N15.1, N30, N34.1, N39.0 |
| Viral hepatitis | ICD-10: B15-B19 |
| Other infections or the sequelae | ICD-10: B90-B99 |
| **Comorbidities** |  |
| Cardiovascular diseases | ICD-8: 393-398, 400-404, 410-414, 427.09, 427.10, 427.19; ICD-10: I05-I09, I10-I15, I20-I25, I50 |
| ACE inhibitors | ATC: C09 (C02 before 1 January 1996) |
| Beta-blockers | ATC: C07 |
| Aspirin | ATC: B01AC06, N02BA01 |
| Clopidogrel | ATC: B01AC04 |
| Statins | ATC: C10AA, B04AB01 |
| Calcium channel antagonists | ATC: C08 |
| Antihypertensive drugs | ATC: C02 |
| Diuretics | ATC: C03 |
| Nitrates | ATC: C01DA (if ≥2 prescriptions are registered). |
| Diabetes | ICD-8: 249, 250; ICD-10: E10, E11, H36.0; ATC codes: A10A, A10B |
| COPD or asthma | ICD-8: 491, 492, 493; ICD-10: J41, J42, J43, J44, J45, J46; ATC: R03 |
| Cancer | ICD-8: 140-209; ICD-10: C00-C99 |
| Fractures or trauma | ICD-8: 800-929, 950-959; ICD-10: S00-T14 |
| Liver disease | ICD-8: 571; ICD-10: K70.0, K70.3, K71.7, K73, K74, K76.0, B18, I85 |
| Obesity | ICD-8: 277; ICD-10: E65-E68 |
| Osteoporosis | ICD-8: 723.09; ICD-10: M80-M82 |
| Pregnancy | ICD-8: 630-680; ICD-10: O00-O99 |
| Renal failure | ICD-8: 581-584; ICD-10: N17-N19 |
| **Antibiotic drugs** |  |
| Penicillins | ATC code: J01C |
| Cephalosporins, aztreonam, carbapenems | ATC code: J01D |
| Macrolides | ATC code: J01FA |
| Quinolones | ATC code: J01M |
| Tetracyclines | ATC code: J01A |
| Sulphonamides | ATC code: J01EB02, J01EE01 |
| **Community antibiotic treatment groups** |  |
| Antibiotics typically used for respiratory tract infection | Macrolides: J01FA |
| Antibiotics typically used for urinary tract infection | Pivampicillin (J01CA02), pivmecillinam (J01CA08), sulphonamide J01EB), sulphonamide with trimethoprim (J01EE), trimethoprim (J01EA), nitrofurantoin (J01XE) |
| Antibiotics typically used for skin or soft tissue infection | Anti-staphylococcal penicillins (J01CF) |
| Focus-unspecific penicillins | J01C (except J01CA02, J01CA08, and J01CF)) |
| **Co-medications** |  |
| Hormone-replacement therapy | G03C, G03F (90 days’ exposure window) |
| NSAIDs | M01AA-M01AH, M01AX01 (60 days’ exposure window) |
| Oral glucocorticoids | H02AB (60 days’ exposure window) |
| Vitamin K antagonists | B01AA03, B01AA04 (90 days’ exposure window) |

| **eTable 2. Incidence rate ratios for venous thromboembolism recorded as primary diagnosis associated with hospital-diagnosed infections and infections treated in the community.** | | | | | | |
| --- | --- | --- | --- | --- | --- | --- |
|  | **Incidence rate ratio (95% confidence intervals)** | | | | | |
|  | **All primary VTE diagnoses** | | | **Unprovoked primary VTE diagnoses** | | |
|  | **No. of cases/controls** | **Unadjusted**^*^ | **Adjusted**^†^ | **No. of cases/controls** | **Unadjusted**^*^ | **Adjusted**^†^ |
| **No infection^‡^** | 5,729/81,610 | 1 (reference) | 1 (reference) | 3,894/46,235 | 1 (reference) | 1 (reference) |
| **Infection, overall^§^** | 3,634/13,479 | 4.0 (3.8–4.2) | 2.6 (2.5–2.7) | 2,052/6,406 | 3.9 (3.6–4.1) | 2.9 (2.7–3.1) |
| **Hospital-diagnosed infection^§^** | 686/992 | 10.1 (8.9–11.4) | 2.8 (2.4–3.2) | 268/287 | 11.2 (9.1–13.9) | 4.2 (3.3–5.4) |
| Respiratory tract infection | 359/367 | 14.8 (12.2–17.9) | 4.4 (3.6–5.5) | 172/123 | 19.4 (14.1–26.6) | 7.6 (5.4–10.8) |
| Urinary tract infection | 172/321 | 7.4 (5.9–9.3) | 1.5 (1.1–2.0) | 66/86 | 7.3 (5.0–10.7) | 1.7 (1.1–2.6) |
| Skin infection | 99/142 | 9.9 (7.3–13.5) | 3.3 (2.3–4.7) | 41/37 | 11.7 (7.0–19.5) | 5.1 (2.8–9.1) |
| Gastrointestinal infection | 92/153 | 9.1 (6.7–12.5) | 2.0 (1.4–3.0) | 16/35 | 5.2 (2.6–10.6) | 2.1 (0.9–4.7) |
| Septicaemia | 52/64 | 12.7 (7.9–20.4) | 3.0 (1.7–5.3) | 13/15 | 10.1 (4.0–25.5) | 3.8 (1.3–11.0) |
| **Community antibiotic treatment**^§^ | 3,337/12,977 | 3.8 (3.6–4.0) | 2.6 (2.5–2.7) | 1,954/6,265 | 3.8 (3.5–4.0) | 2.9 (2.7–3.1) |
| Antibiotics for respiratory tract infection | 707/2,213 | 4.9 (4.5–5.4) | 3.3 (2.9–3.7) | 427/1,095 | 5.0 (4.4–5.7) | 3.5 (3.0–4.1) |
| Antibiotics for urinary tract infection | 1,061/5,061 | 3.3 (3.0–3.5) | 1.9 (1.7–2.0) | 554/2,311 | 2.9 (2.6–3.2) | 2.0 (1.8–2.3) |
| Antibiotics for skin or soft tissue infection | 478/1,028 | 7.1 (6.3–8.1) | 3.9 (3.3–4.5) | 253/409 | 7.8 (6.5–9.5) | 5.8 (4.7–7.1) |
| Focus-unspecific penicillins | 1,761/6,007 | 4.3 (4.0–4.5) | 3.1 (2.8–3.3) | 1,108/3,009 | 4.5 (4.1–4.9) | 3.5 (3.2–3.9) |

^*^Age-, gender-, and county-matched conditional logistic regression.

^†^Adjusted for the classical VTE risk factors, other comorbidities, another recent hospital admission and co-medications use, as listed in Table 1. Classical risk factors were not included, per definition, in the model for unprovoked VTE.

^‡^No hospital-diagnosed infection or filled community antibiotic prescription within 365 days before the VTE.

^§^In- or outpatient hospital-diagnosed infection and/or filled community antibiotic prescription within 3 months before the VTE.

| **eTable 3. Incidence rate ratios for infection associated with deep venous thrombosis or pulmonary embolism.** | | | | | | |
| --- | --- | --- | --- | --- | --- | --- |
|  | **Incidence rate ratio (95% confidence interval)** | | | | | |
|  | **Deep venous thrombosis** | | | **Pulmonary embolism** | | |
|  | **Cases/controls** | **Unadjusted*** | **Adjusted**^†^ | **Cases/controls** | **Unadjusted*** | **Adjusted**^†^ |
| **No infection^‡^** | 4,739/67,221 | 1 (reference) | 1 (reference) | 2,349/36,149 | 1 (reference) | 1 (reference) |
| **Infection, overall**^§^ | 2,900/11,005 | 3.9 (3.7–4.1) | 2.5 (2.4–2.7) | 1,936/6,362 | 4.9 (4.6–5.3) | 2.9 (2.7–3.1) |
| **Hospital-diagnosed infection**^§^ | 569/754 | 11.3 (9.8–13.0) | 3.1 (2.6–3.6) | 493/550 | 14.3 (12.2–16.8) | 3.6 (2.9–4.3) |
| Respiratory tract infection | 258/270 | 13.9 (11.2–17.3) | 4.1 (3.2–5.3) | 303/229 | 22.1 (17.4–28.0) | 5.9 (4.4–7.8) |
| Urinary tract infection | 149/214 | 11.0 (8.4–14.5) | 2.5 (1.8–3.4) | 94/200 | 6.8 (5.1–9.2) | 1.1 (0.7–1.5) |
| Skin infection | 118/114 | 14.6 (10.6–20.0) | 5.0 (3.5–7.3) | 27/57 | 8.4 (4.8–14.6) | 2.0 (1.0–4.0) |
| Intra-abdominal infection | 83/135 | 9.6 (6.9–13.5) | 2.2 (1.5–3.3) | 64/77 | 12.0 (8.0–18.1) | 2.7 (1.7–4.5) |
| Septicaemia | 61/39 | 20.5 (12.2–34.4) | 4.1 (2.3–7.5) | 37/42 | 17.0 (9.3–31.1) | 3.2 (1.5–6.8) |
| **Antibiotic treatment**^§^ | 2,635/10,601 | 3.7 (3.5–3.9) | 2.5 (2.4–2.7) | 1,721/6,092 | 4.5 (4.2–4.9) | 2.8 (2.6–3.1) |
| Antibiotics for respiratory tract infection | 477/1,826 | 4.0 (3.5–4.5) | 2.6 (2.3–3.0) | 462/982 | 7.7 (6.7–8.9) | 5.0 (4.2–5.8) |
| Antibiotics for urinary tract infection | 845/4,006 | 3.2 (3.0–3.5) | 1.9 (1.7–2.1) | 586/2624 | 3.7 (3.3–4.2) | 2.0 (1.7–2.3) |
| Antibiotics for skin or soft tissue infection | 467/857 | 8.2 (7.1–9.4) | 4.7 (4.0–5.6) | 148/470 | 5.2 (4.2–6.5) | 2.1 (1.6–2.8) |
| Focus-unspecific penicillins | 1,375/4,969 | 4.0 (3.7–4.3) | 2.8 (2.6–3.1) | 891/2,667 | 5.3 (4.9–5.9) | 3.6 (3.2–4.0) |

^*^Age-, gender-, and county-matched conditional logistic regression.

^†^Adjusted for the classical VTE risk factors, other comorbidities, another recent hospital admission and co-medication use, as listed in Table 1.

^‡^No hospital-diagnosed infection or filled community antibiotic prescription within 365 days before the VTE.

^§^In- or outpatient hospital-diagnosed infection and/or filled community antibiotic prescription within 3 months before the VTE.

| **eTable 4. Incidence rate ratios for infection associated with venous thromboembolism diagnosed in settings other than the emergency room.** | | | | |
| --- | --- | --- | --- | --- |
|  | **Incidence rate ratio (95% confidence intervals)** | | | |
|  | **All VTE** | | **Unprovoked VTE** | |
|  | **Unadjusted**^*^ | **Adjusted**^†^ | **Unadjusted**^*^ | **Adjusted**^†^ |
| **No infection^‡^** | 1 (reference) | 1 (reference) | 1 (reference) | 1 (reference) |
| **Infection, overall^§^** | 4.3 (4.1–4.5) | 2.7 (2.6–2.8) | 4.1 (3.8–4.3) | 3.0 (2.8–3.2) |
| **Hospital-diagnosed infection^§^** | 12.8 (11.5–14.3) | 3.4 (3.0–3.9) | 13.9 (11.5–16.8) | 5.0 (4.0–6.3) |
| Respiratory tract infection | 17.9 (15.2–21.1) | 5.1 (4.2–6.1) | 20.5 (15.6–26.9) | 7.5 (5.6–10.2) |
| Urinary tract infection | 8.9 (7.3–10.9) | 1.7 (1.4–2.2) | 8.8 (6.2–12.5) | 2.0 (1.3–3.0) |
| Skin infection | 12.9 (9.8–17.1) | 4.2 (3.0–5.9) | 13.2 (8.2–21.2) | 5.7 (3.3–9.7) |
| Intra-abdominal infection | 10.6 (8.2–13.8) | 2.5 (1.8–3.4) | 9.3 (5.1–17.1) | 3.4 (1.6–6.9) |
| Septicaemia | 19.5 (13.1–29.1) | 3.8 (2.4–6.1) | 13.3 (5.8–30.6) | 4.8 (1.8–12.4) |
| **Antibiotic treatment**^§^ | 4.0 (3.9–4.2) | 2.7 (2.5–2.8) | 3.9 (3.7–4.2) | 3.0 (2.8–3.2) |
| Antibiotics for respiratory tract infection | 5.3 (4.9–5.8) | 3.5 (3.1–3.8) | 5.3 (4.7–6.0) | 3.7 (3.2–4.2) |
| Antibiotics for urinary tract infection | 3.5 (3.3–3.8) | 2.0 (1.9–2.2) | 3.2 (2.9–3.5) | 2.2 (2.0–2.4) |
| Antibiotics for skin or soft tissue infection | 7.1 (6.3–8.1) | 3.8 (3.3–4.4) | 7.4 (6.2–8.8) | 5.5 (4.6–6.7) |
| Focus-unspecific penicillins | 4.5 (4.2–4.8) | 3.1 (2.9–3.3) | 4.7 (4.3–5.0) | 3.6 (3.3–4.0) |

^*^Age-, gender-, and county-matched conditional logistic regression.

^†^Adjusted for the classical risk factors, other comorbidities, another recent hospital admission and co-medication use, as listed in Table 1. Classical risk factors were not included, per definition, in the model for unprovoked VTE.

^‡^No antibiotic prescription redemption or hospital infection diagnosis within 365 days before the VTE.

^§^In- or outpatient hospital-diagnosed infection and/or filled antibiotic prescription within 3 months before the VTE.
